# Primary angiosarcoma of breast in an octogenarian woman: A case report with literature review

**DOI:** 10.1016/j.ijscr.2023.108163

**Published:** 2023-04-11

**Authors:** Lubna M. Vohra, Dua Jabeen, Romana Idrees

**Affiliations:** a Department of Breast Surgery, Aga Khan University Hospital, Karachi, Pakistan; b Department of Medical Oncology, Aga Khan University Hospital, Karachi, Pakistan; c Department of Pathology, Aga Khan University Hospital, Karachi, Pakistan

**Keywords:** Angiosarcoma, Breast, Case reports, Mastectomy, Prognosis

## Abstract

**Introduction and importance:**

Primary breast angiosarcomas are endothelial derived breast sarcomas found in younger to middle age groups. The diagnosis of primary breast angiosarcoma in an octogenarian woman is a rare incidence.

**Case presentation:**

We report a case of 87-year-old postmenopausal woman presenting with history of lump in her right breast for four months. Ultrasound guided biopsy was performed which confirmed the diagnosis of angiosarcoma hence, subjected to simple mastectomy. She was doing quite well for one year when ultimately, she developed metastatic disease and couldn't survive more.

**Clinical discussion:**

Microscopically, these tumours are classified into grades I, II and III. Hematogenous route has been taken for metastasis having lungs being most involved. There are limited case reports and studies that have investigated the use of adjuvant radio/chemotherapy.

**Conclusion:**

Primary angiosarcoma of breast is a rare disease in old age group with limited treatment options which resulted in poor prognosis and early relapse.

## Highlights

• Primary angiosarcoma of breasts have a tendency to grow rapidly with early hematogenous spread.
• We are reporting a case of primary breast angiosarcoma in a woman who is in her eight decade of life which is a rare occurrence for age.
• There are limited treatment options available in older age group to avoid relapse.

## 1 Introduction

Breast sarcomas are rare and exceptional non-epithelial tumours having soft tissue as their cell of origin in the breast. They possess a natural behavior, management, and treatment options that is different from breast carcinomas. Among these, breast angiosarcomas run an extremely aggressive course [1]. They are usually encountered in women who are in there second to fourth decades of life. Despite of being infrequently discovered, they are the most common diagnosed type of breast sarcomas making up almost 8 % of their cases and 0.04 % of all malignant breast lesions [2]. Here, we present a case of primary breast angiosarcoma explored in a woman who is in her eight decade of life. To the best of our knowledge, only one case has been reported in this age group in the available literature which compelled us to write the following case report [3]. The SCARE guidelines were duly followed in preparation of this case report [4].

## 2 Presentation of case

This is a case report of 87-year-old postmenopausal woman, known hypertensive, who was diagnosed with Primary Angiosarcoma of breast in July 2021: The lady noticed lump in right breast for four months however she never visited doctor for this complaint, subsequently she noticed gradual increase in size hence visited primary physician who subjected breast to Ultrasound and subsequent biopsy which showed atypical endothelial proliferation and CD34 positivity raising the possibility of vascular neoplastic lesion.

**Fig. 1:**
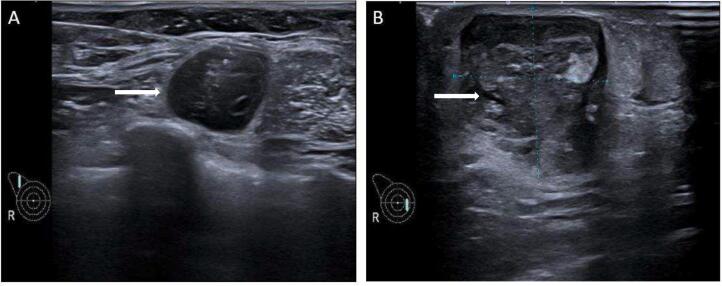
Ultrasound showed two lobulated, hypoechoic, solid lesions (white arrows) in right breast. (A) Near axillary tail (B) Lower inner quadrant.

**Fig. 2:**
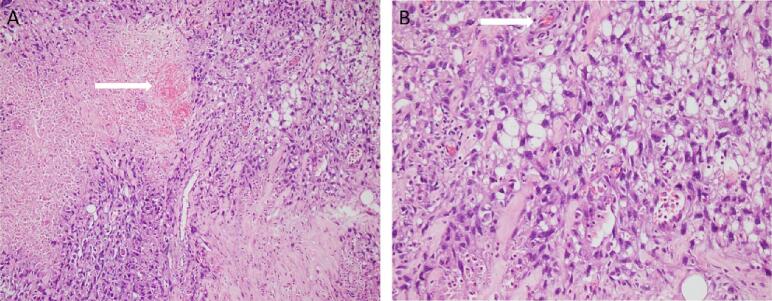
Photomicrograph showing atypical endothelial cells lining anastomosing network of vascular channels as shown by white arrows (haematoxylin and eosin stain). (A) Low power field (B) High power field.

After two months of biopsy, she visited breast clinic and clinical examination identified a soft 2.5 × 2.5 cm mobile lump in lower inner quadrant of right breast and another 1.5 cm similar morphology lump close to axillary tail. She was subjected to Mammogram and Ultrasound which identified two lobulated, hypoechoic, solid lesions with significant vascularity (Fig. 1 A, B). Ultrasound guided biopsy confirmed both as Primary Angiosarcoma of breast. There was no history of any malignancy in her family. CT scan of whole abdomen and pelvis was then performed which showed no evidence of metastatic disease hence after discussion in Multidisciplinary team (MDT) subjected to mastectomy. The mastectomy specimen identified two tan brown to grey, white hemorrhagic lesions one in lower inner quadrant 3 × 2.5 × 2 cm and another at axillary tail 2 × 1.8.1.3 cm. This shows malignant vascular neoplastic lesion characterized by anastomotic vascular channels lined by atypical endothelial cells which appears spindly, frequent mitotic figures seen (Fig. 2A, B).

**Fig. 3:**
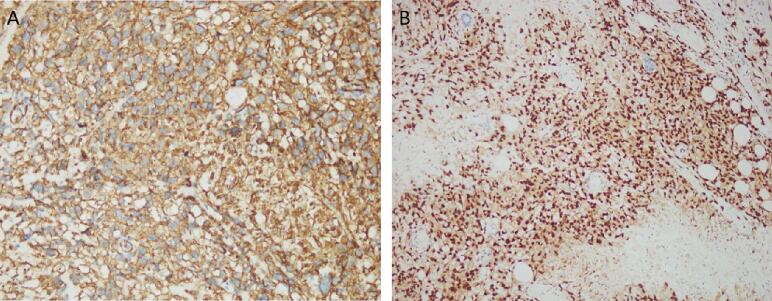
Immunohistochemical stains revealed tumor cells positive for: (A) CD34 (B) ERG.

Immunohistochemical stains were performed which showed following reactive pattern: CD34 positive, ERG positive, CAM5.2 negative (Fig. 3A, B).

The case was discussed in MDT and consensus was to keep her under surveillance only. Patient was followed every three months by Primary physician regularly. Subsequently she developed bilateral pleural effusion and widespread metastatic disease in October 2022, core biopsy of one of the pulmonary nodule was performed which confirmed to have secondaries from primary disease. She was then kept in palliative care and succumbed to death after 25 days.

## 3 Discussion

Angiosarcomas are rare distinct entity of breast malignant tumours that are derived from vascular endothelial cells. They can be stratified into primary and secondary lesions, the latter being less common than primary and occur because of prior breast radiations. Breast angiosarcomas may have an initially clinical silent course presenting usually as a painless palpable mass that may enlarge rapidly. Other clinical manifestations varies from purplish or eczematous rash, hematoma formation to diffuse breast enlargement [5], [6]. Male breasts are not exempted from these type of tumours and up to sixth cases have been present in the published data [7].

Histologically, angiosarcomas are differentiated into grades I, II and III. Grade I (well-differentiated) tumours resemble benign vascular lesions consisting of anastomosing, often dilated pattern of channels lined by minimally atypical endothelial cells with plump, hyperchromatic nuclei. Mitosis and tufting of endothelial cells is rarely seen. An infiltrative growth pattern distinguished it from benign vascular lesions. Grade II/ moderately differentiated tumours are identical to well differentiated tumours but with increased mitoses, endothelial tufting, and foci of solid growth pattern. Grade III (poorly differentiated) exhibit marked pleomorphism, mitoses, necrosis, and solid growth. They may show formation of blood lakes due to the extravasation of blood from malignant vessels. Epithelioid and spindled cytology with no obvious vasoformative morphology can also be appreciated on microscopy [8], [9].

Angiosarcomas have a propensity to spread early and aggressively through hematogenous route with a reported range of 16–44 %. Lungs remain the most frequent site of metastasis followed by liver, bone, and skin [10], [11].

Treatment of angiosarcomas is a challenging situation for multidisciplinary teams including surgeons and medical and radiation oncologists. Total mastectomy remains the standard therapeutic surgical operation in breast angiosarcomas. Since node metastasis are rare, axillary dissection is spared unless there is clinical evidence of lymphatic involvement [12]. Postoperative radiation is recommended for microscopically positive margins following surgical resection. The role of Adjuvant chemotherapy is a matter of debate but published data has shown that it improves overall survival rates in those with localized and metastatic disease [13]. The most common chemotherapeutic regimens include doxorubicin, adriamycin, ifosfamide, cyclophosphamide, vincristine, and paclitaxel, typically administered weekly [14]. Pervaiz et al. revealed the significance of adjuvant doxorubicin-based chemotherapy in prolonging local and distant recurrence and improving the overall recurrence-free survival [15]. Taxane based chemotherapy in metastatic angiosarcoma demonstrated an increment in overall survival from 10.4 to 23.7 months [16]. Targeted therapy against vascular endothelial growth factor (VEGFR/VEGF pathway) has also been explored but clinical trials have shown limited survival rates in comparison to chemotherapeutic agents [14].

Angiosarcoma has a very dismal prognosis of all breast sarcomas, with median recurrence free survival <3 years and overall survival <6 years [9]. Better prognosis seen with non-menopausal status, negative surgical margins, lack of tumor necrosis, low or intermediate grade lesions [17]. The 5 years disease-free survival rate for grade I, II, and III tumours was 76 %, 70 %, and 15 % respectively [18]. Some studies have indicated tumor size carrying some prognostic value while others have analyzed no association between size and survival [17], [19].

## 4 Conclusion

The occurrence of primary breast angiosarcoma in an elderly woman is a rare phenomenon warranting accurate and timely diagnosis due to the fast-growing and metastatic nature of disease. These tumours are notorious for having worst prognosis than other breast sarcomas. Complete surgical excision has been the gold standard treatment of breast angiosarcomas. There is a paucity of data supporting the role of radiotherapy and adjuvant chemotherapy in this rare disease to prevent recurrences and systemic spread.

## Patient's consent

Written informed consent was obtained from the patient's next of kin for publication of this case report and accompanying images. A copy of the written consent is available for review by the.

Editor-in-Chief of this journal on request.

## Provenance and peer review

Not commissioned, externally peer reviewed.

## Ethical approval

Ethical approval was provided by the authors institution.

## Funding

The authors have no funding resources to be declared.

## Author contribution

Conceptualization, Methodology: LMV, DJ, RI.

Writing - Original Draft: LMV, DJ.

Writing - Review & Editing: All authors.

Final approval of the Article: All authors.

Accountability for all aspects of the work: All authors.

## Guarantor

Lubna M. Vohra.

Assistant Professor

Consultant Breast Surgeon.

lubna_mushtaque@hotmail.com.

## Conflict of interest statement

The authors have no conflicts of interest to declare.

## References

[1] Duncan M.A., Lautner M.A. (2018 Aug). Sarcomas of the breast. Surg. Clin. N. Am..

[2] Bhosale S.J., Kshirsagar A.Y., Patil M.V., Wader J.V., Nangare N., Patil P.P. (2013). Primary angiosarcoma of breast: a case report. Int. J. Surg. Case Rep..

[3] Athar M., Singh S., Chaudhary A.K., Sachan M., Khan L., Jamal S. (2017). Primary angiosarcoma of the breast in a seventy–year–old female: a rare case report. Ann. Res. Hosp..

[4] Agha R.A., Franchi T., Sohrabi C., Mathew G., Kerwan A., SCARE Group (2020 Dec). The SCARE 2020 guideline: updating consensus Surgical CAse REport (SCARE) guidelines. Int. J. Surg..

[5] Arora T.K., Terracina K.P., Soong J., Idowu M.O., Takabe K. (2014 Feb). Primary and secondary angiosarcoma of the breast. Gland Surg..

[6] Pang F.T., Lee S.Y., Kaur M. (2021 Dec). Primary breast angiosarcoma: a case report. Egypt. J. Radiol. Nucl. Med..

[7] da Silva B.B., Eulálio Filho W.M.N., Costa P.V.L., Silva R.A., Junior A.M.C., Chagas D.C., de Almeida Melo M., Neto F.M., Tavares C.B., de Sousa Júnior E.C., Coelho E.G., Campelo V., Gebrim L.H., da Silva Junior R.G. (2018 Oct 16). A rare case of primary breast angiosarcoma in a male: a case report. BMC Cancer.

[8] Bennani A., Chbani L., Lamchahab M., Wahbi M., Alaoui F.F., Badioui I., Melhouf M.A., Amarti A. (2013 Apr). Primary angiosarcoma of the breast: a case report. Diagn. Pathol..

[9] ES Reisenbichler . Angiosarcoma. PathologyOutlines.com website. https://www.pathologyoutlines.com/topic/breastmalignantangiosarcoma.html. Accessed February 7th, 2023.

[10] Gaballah A.H., Jensen C.T., Palmquist S., Pickhardt P.J., Duran A., Broering G., Elsayes K.M. (2017 Jul). Angiosarcoma: clinical and imaging features from head to toe. Br. J. Radiol..

[11] Chen K.T., Kirkegaard D.D., Bocian J.J. (1980 Jul 15). Angiosarcoma of the breast. Cancer.

[12] Wu M., Huang Y., Tian W., Yao Y., Deng Y. (2019 Oct). Recurrence of primary breast angiosarcoma 7 years after mastectomy in a 17-year-old woman: a case report. Breast Care (Basel).

[13] Bordoni D., Bolletta E., Falco G., Cadenelli P., Rocco N., Tessone A., Guarino S., Accurso A., Amato B., Magalotti C. (2016). Primary angiosarcoma of the breast. Int. J. Surg. Case Rep..

[14] Esposito E., Avino F., di Giacomo R., Donzelli I., Marone U., Melucci M.T., Rinaldo C., Ruffolo F., Saponara R., Siani C., Tortoriello R., Botti G., Rinaldo M., Fucito A. (2019 Oct). Angiosarcoma of the breast, the unknown-a review of the current literature. Transl. Cancer Res..

[15] Pervaiz N., Colterjohn N., Farrokhyar F., Tozer R., Figueredo A., Ghert M. (2008 Aug 1). A systematic meta-analysis of randomized controlled trials of adjuvant chemotherapy for localized resectable soft-tissue sarcoma. Cancer.

[16] Hirata T., Yonemori K., Ando M., Hirakawa A., Tsuda H., Hasegawa T., Chuman H., Namikawa K., Yamazaki N., Fujiwara Y. (2011 Jul-Aug). Efficacy of taxane regimens in patients with metastatic angiosarcoma. Eur. J. Dermatol..

[17] Bousquet G., Confavreux C., Magné N., de Lara C.T., Poortmans P., Senkus E., de Lafontan B., Bolla M., Largillier R., Lagneau E., Kadish S., Lemanski C., Ozsahin M., Belkacémi Y. (2007 Dec). Outcome and prognostic factors in breast sarcoma: a multicenter study from the rare cancer network. Radiother. Oncol..

[18] Rosen P.P., Kimmel M., Ernsberger D. (1988 Nov 15). Mammary angiosarcoma. The prognostic significance of tumor differentiation. Cancer.

[19] Zelek L., Llombart-Cussac A., Terrier P., Pivot X., Guinebretiere J.M., Le Pechoux C., Tursz T., Rochard F., Spielmann M., Le Cesne A. (2003 Jul 1). Prognostic factors in primary breast sarcomas: a series of patients with long-term follow-up. J. Clin. Oncol..

